# Clinical usefulness of anti-α3Gal immunoglobulin E assays for cetuximab-mediated anaphylaxis in head and neck cancer

**DOI:** 10.1016/j.iotech.2025.101041

**Published:** 2025-02-07

**Authors:** Y. Pointreau, C. Freneaux, T. Bejan-Angoulvant, D. Ternant, G. Calais, H. Watier

**Affiliations:** 1 CHRU de Tours, Service de radiothérapie, Tours, France; 2 Université de Tours, Tours, France; 3 Institut inter-régional de Cancérologie, Centre Jean Bernard - Clinique Victor Hugo, Centre de Cancérologie de La Sarthe, Le Mans, France; 4 CHRU de Tours, Service d’immunologie, Tours, France; 5 CHRU de Tours, Service de pharmacologie médicale, Tours, France

**Keywords:** cetuximab, infusion reaction, hypersensitivity assays, head and neck cancer, anti-α3Gal IgE

## Abstract

**Background:**

Fatal anaphylactic reactions to cetuximab remain a clinical issue, although they are associated with preexisting immunoglobulin E (IgE) directed against the galactose-α1,3-galactose epitope (α3Gal). We aimed to compare the clinical usefulness of the two assays and determine the prevalence of preexisting anti-α3Gal IgE.

**Patients and methods:**

An anti-α3Gal IgE assay was developed (70BP assay) and compared with a commercial assay [bovine thyroglobulin (bTG) assay]. Both assays were applied to two cohorts: 299 healthy blood donors and 41 patients with head and neck cancer treated with cetuximab, including four patients with a history of anaphylactic reaction (9.8%).

**Results:**

The prevalence of anti-α3Gal IgE was 6% and 5% using 70BP and bTG assays, respectively, in healthy blood donors. Among the head and neck cancer patients, the four who had an anaphylactic reaction were included in the seven (17.1%) and six (14.6%) patients with a signal above the detection threshold using the 70BP and bTG assays, respectively. This resulted in a sensitivity and negative predictive value of 100% for both assays, with a specificity of 91.9% and 94.6%, respectively, and a positive predictive value of 57.1% and 66.6% for the 70BP and bTG assays, respectively. Using an optimized threshold in the bTG assay, the prevalence of anti-α3Gal IgE in blood donors decreased to 1.3%, and five patients (12.2%) were eventually considered positive, giving a specificity of 97.3% and a positive predictive value of 80%.

**Conclusion:**

The predictive value of anti-α3Gal IgE using these two assays was excellent and useful in clinical practice.

## Highlights

• Use of cetuximab combined with radiotherapy or chemotherapy were standard of care in head and neck cancer.
• Hypersensitivity reactions to cetuximab could be fatal due to anaphylactic reactions.
• Measurement of anti-alpha-Gal IgE levels before prescribing cetuximab is recommended to predict infusion reaction risks.
• In case of negative results (<0.1 kAU/l level using bTG assay), cetuximab can be safely used in patients with head and neck cancer.
• In case of positive results (>0.1 kAU/l level using bTG assay), more data are needed to ensure the safe use of cetuximab.

## Introduction

For over 30 years, >30 monoclonal antibodies have been approved for cancer treatment, all displaying a favorable benefit-to-risk ratio despite frequent adverse effects, particularly infusion reactions.^1^ Anaphylaxis is mostly due to a single agent, cetuximab (Erbitux, ImClone–Eli Lilly and Merck), with potentially fatal outcomes.^2^^,^^3^

Cetuximab is a chimeric human-mouse immunoglobulin (Ig) G1 kappa monoclonal antibody directed against the extracellular ligand-binding domain of the epidermal growth factor receptor. It is approved for locally advanced head and neck squamous cell carcinoma in association with radiotherapy,^4^^,^^5^ for recurrent or metastatic head and neck squamous cell carcinoma in association with chemotherapy,^6^ and for metastatic colorectal cancer alone or in combination with chemotherapy.^7^, ^8^, ^9^, ^10^, ^11^ The addition of cetuximab to the treatment of these cancers has improved overall and/or progression-free survival.^5^^,^^10^

In locally advanced head and neck cancer treated with radiotherapy, cetuximab is the only approved monoclonal antibody for patients not eligible for platinum-based chemotherapy, as are other alternatives such as panitumumab, bevacizumab, or aflibercept for metastatic colorectal cancers. Some studies have shown that infusion reactions during the first infusion of cetuximab occur more frequently in patients with head and neck cancer than in patients with colorectal cancer.^2^ Therefore it is of utmost importance to ensure the safe use of cetuximab in patients with head and neck cancer.

Hypersensitivity reactions to cetuximab usually occur within seconds or minutes after the patient’s first exposure^2^^,^^12^ and are related to the presence of preexisting anti-cetuximab IgE, as reported in a landmark study.^12^ The incidence of severe hypersensitivity reactions to cetuximab has been reported to range from 3% to 22% of patients.^9^^,^^13^, ^14^, ^15^, ^16^ Environmental factors, such as tick bites, have been proposed to explain the variable proportions of individuals with preexisting anti-cetuximab IgE^17^ and the highly variable incidence of anaphylactic reactions to cetuximab across studies and geographic areas.^9^^,^^13^^,^^17^^,^^18^ A history of atopy, age, sex, ethnicity, smoking, and additional therapies (e.g. chemotherapy or radiotherapy) have also been proposed as factors favoring anaphylaxis to cetuximab, although their roles remain controversial.^13^^,^^15^^,^^18^

**Figure 1:**
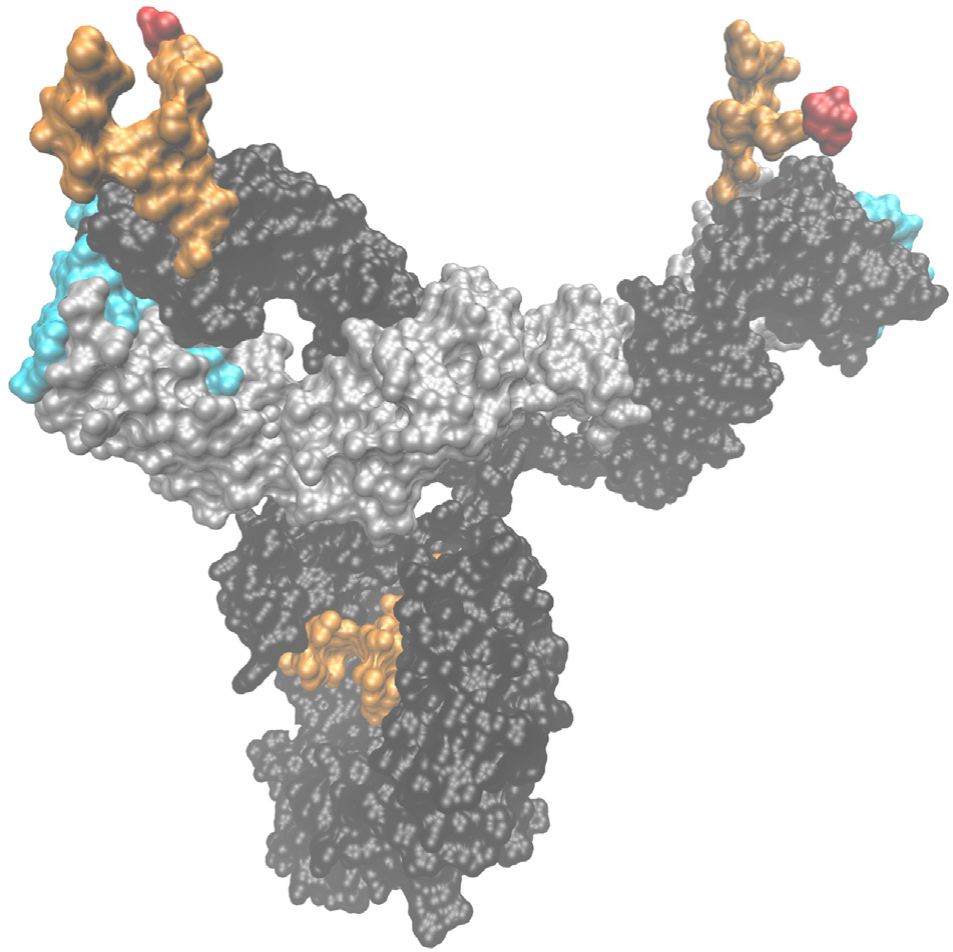
**Tridimensional model of an entire molecule of cetuximab with its Fc- and VH-associated biantennary *N*-glycans (orange); the antennae of the VH-associated *N*-glycans are terminated with α3Gal residues (in red).** This surface representation has been built with the VMD software (University of Illinois), starting from the structure of the only available entire human immunoglobulin G1 kappa monoclonal antibody (anti-HIV-1 gp120 clone b12, pdb #1hzh) with the κ light and γ1 heavy chains in gray and black, respectively. The two Fc *N*-glycans are depicted in orange. Its Fab fragments were replaced by those of cetuximab (pdb #1yy9).^25^ The CDR residues from the light and heavy chains forming the antigen-binding sites are shown in cyan. The crystalized cetuximab Fab fragments, having been produced in mammalian cells, harbor the VH *N*-glycan; however, only the first GlcNAc residue was resolved because the remaining part of the glycan was too mobile to adopt a unique conformation (pdb #1yy9).^25^ To visualize a given conformation of these VH *N*-glycans, an Fc-derived digalactosylated *N*-glycan of pdb #1hzh was superimposed on the GlcNAc residue and represented in orange. Finally, to represent an α3Gal residue (in red) ending one of the arms of the biantennary glycans, as commonly observed in cetuximab, a Galα3Galβ4GlcNAcβtrisaccharid originally crystallized in *Clostridium difficile* A toxin (pdb #2G7) was superimposed to the corresponding arms of the VH *N*-glycans.

Anti-cetuximab IgE is specific to an oligosaccharide, galactose-α1,3-galactose (α3Gal), terminating an *N*-glycan present in the variable domain of the cetuximab heavy chains (Figure 1).^13^^,^^19^, ^20^, ^21^ Depending on the tested batches and methods used, 62%-75% of cetuximab Fab *N*-glycans carry at least one α3Gal-terminated antenna (Figure 1).^19^, ^20^, ^21^ Therefore 38%-56% of cetuximab molecules could expose very accessible α3Gal residues on their two Fab arms (Figure 1),^22^ potentially explaining their ability to bridge mastocyte-bound anti-α3Gal IgE and trigger an anaphylactic reaction, a feature not shared by other biopharmaceuticals expressing isolated α3Gal-containing *N*-glycans.^23^^,^^24^

The risk of fatal anaphylactic reactions using cetuximab is so concerning that an international congress was held in France to discuss practical measures to be taken.^26^ We also emphasized the need for accurate assays^27^ following a published report in the journal of clinical oncology with a fatal infusion reaction to cetuximab.^3^ Following the initially described assay,^28^ Mariotte et al.^29^ proposed an enzyme-linked immunosorbent assay based on IgE binding to the Fab fragment of cetuximab. Using this assay, anti-cetuximab IgE was frequently detected in blood donors and patients with cancer (28.2% and 26.1%, respectively), although only 8.7% of grade III hypersensitivity reactions were observed in treated patients. Despite a very good negative predictive value (98.5%), the low positive predictive value (33.3%) of the assay could lead to an overdiagnosis of the putative risk of cetuximab-induced anaphylaxis. This overdiagnosis may lead to decreased survival of patients with cancer if cetuximab is erroneously contraindicated, particularly in locally advanced head and neck cancer when no therapeutic alternative currently exists.

We have developed an alternative assay based on IgE binding to α3Gal glycans (70BP assay) and compared it with Thermo Fisher Scientific’s bovine thyroglobulin assay (bTG assay)^26^ to detect red meat allergy in patients. This assay is based on IgE binding to bTG, which is known to be heavily α-galactosylated.^30^

We aimed to compare the prevalence and associated factors of anti-α3Gal IgE using the 70BP and bTG assays in a cohort of healthy blood donors and in patients with head and neck cancer treated with cetuximab and to determine their predictive values for anaphylaxis in cetuximab-treated patients.

## Patients and methods

### Patients

Two local cohorts were included in this study. The first cohort contained sera from 299 healthy anonymous blood donors. The only available characteristics were age, sex, and ABO blood group. The second cohort included 41 consecutive patients with head and neck cancer who were cared for and followed up in the radiotherapy unit of the Henry-Kaplan Centre at the University Hospital Centre of Tours. All the patients received cetuximab plus radiotherapy or chemotherapy. Before cetuximab infusion, patients received premedication with corticosteroids and antihistamine drugs as recommended.^31^ Data prospectively collected included age, sex, history of allergy (e.g. drugs, food, animals), tobacco and alcohol use, and occurrence of a previous hypersensitivity reaction. The grading of hypersensitivity reactions was based on the Common Terminology Criteria for Adverse Events (CTCAE) version 4.03.^32^ This grading scale ranges from grade 3, indicating symptomatic bronchospasm (with or without urticaria), requiring parenteral intervention, allergy-related edema/angioedema, or hypotension; to grade 4, involving life-threatening consequences requiring urgent intervention; and grade 5, indicating death.

### Helsinki compliance

Human investigations were carried out after approval by the local Human Investigations Committee and in accordance with an assurance filed with and approved by the Department of Health and Human Services. The study adhered to the ethical standards of the institutional committee and the 1964 Declaration of Helsinki and its later amendments. The study complies with the reference methodology adopted by the French Data Protection Authority (CNIL), and patients, or their proxies, were informed about the study in writing and provided no objections to the use of their clinical data for research purposes. All patients were informed of the study protocol. The investigators requested the use of the patients’ medical data for this study. Patients were given sufficient opportunity to refuse access to their data; in the absence of a response, nondeclarative consent was assumed. The patients were also reminded that their data would be anonymized and that the analyses would be confidential.

### Serum samples

Pretreatment serum samples were routinely collected from each patient before cetuximab infusion. In the two cohorts, the total IgE concentration and ImmunoCAP Phadiatop (routinely used to detect specific IgE against common aeroallergens) were determined as atopic biological markers. Tryptase levels were measured in patients who developed an anaphylactic reaction to cetuximab.

### Immunoassays

The anti-α3Gal IgE was measured using two assays. Both assays were based on the ImmunoCAP system (Phadia Thermo Fischer Scientific).

#### Setting up the 70BP assay

As the α3Gal epitope is the only xenogeneic cetuximab antigen explaining preexisting (natural) IgE,^28^ we decided to set up our assay based on synthetic α-galactosylated antigens (Supplementary Figure S1, available at https://doi.org/10.1016/j.iotech.2025.101041). Preliminary experiments were carried out with sera collected from three patients with grade 3 or 4 reactions during the first cetuximab infusion, hypothesizing that their sera contained anti-α3Gal IgE.

**Figure 2:**
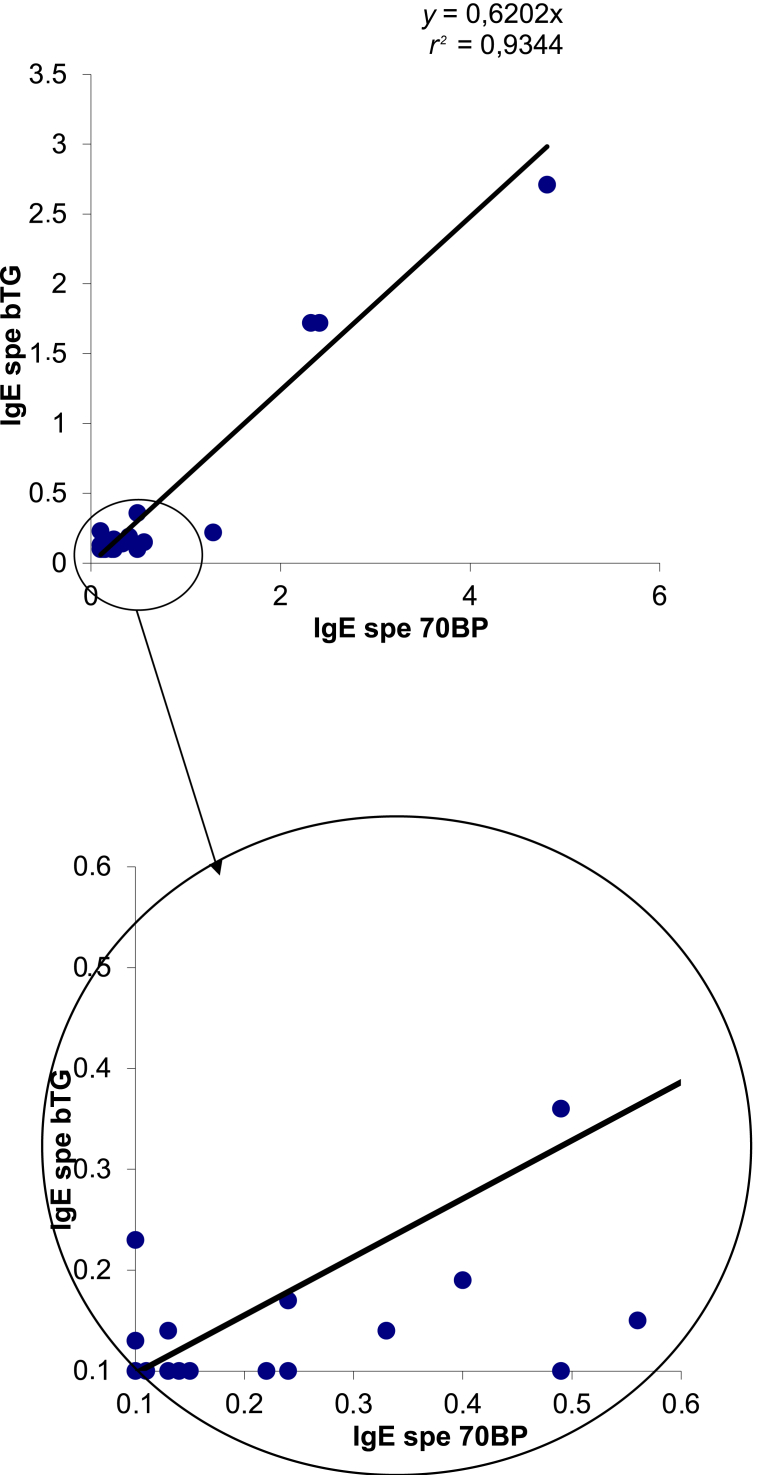
**Correlation between the two assays (70BP and bTG) to detect the presence of immunoglobulin E (IgE) in the sera of 299 blood donors (*r***^**2**^**= 0.91).** bTG, bovine thyroglobulin.

To optimize the detection of anti-α3Gal IgE in our assay, we tested a panel of different α-galactosylated antigens from Lectinity (Moscow, Russia), containing either α3Gal or α4Gal terminal residues (Supplementary Figure S1, available at https://doi.org/10.1016/j.iotech.2025.101041). α4Gal oligosaccharides were chosen as negative controls because they do not bind to anti-α3Gal antibodies.^33^ All tested antigens were biotinylated to allow their capture by the streptavidin-coated solid phase. They were used either in monomeric (70BM for α3Gal and 8BM for α4Gal) or in polymeric forms with four trisaccharides bound to poly[*N*-(2-hydroxyethyl)acrylamide], as shown in Figure 2. The polymeric antigens were based either on *N*-acetyllactosamine (70BP for α3Gal and 8BP for α4Gal) or on a lactose structure (10BP for α3Gal and 37BP for α4Gal) (Supplementary Figure S1, available at https://doi.org/10.1016/j.iotech.2025.101041).

Experiments were conducted using an ImmunoCAP 100 (Thermo Fischer Scientific) instrument with the appropriate reagents, following the manufacturer’s instructions. Streptavidin-coated ImmunoCAPs (Thermo Fischer Scientific) were incubated with 50 μl of the different biotinylated and α-galactosylated antigens at different dosages (1-33.33 μg) in phosphate-buffered saline (PBS) solution for 30 min at 37°C and washed. Fifty microliters of serum were then added for 30 min at 37°C, and the systems were washed again. To assess the specificity of detection, a negative control was carried out using antigen-free wells containing PBS only. Bound IgE was detected using an anti-human IgE (50 μl) mouse monoclonal antibody coupled to β-galactosidase (Thermo Fischer Scientific) and incubated for 24 min at 37°C. After washing, 50 μl enzyme substrate (4-methylumbelliferyl-β-d-galactoside) was added. The fluorescence signal was converted into arbitrary units (kAU/l) using a total IgE calibration curve, with a detection threshold of 0.1 kAU/l.

The three sera were tested against three α3-galactosylated antigens (70BM, 70BP, and 10BP) and in parallel with their α4 counterparts (8BM, 8BP, and 37BP, respectively; Supplementary Table S1, available at https://doi.org/10.1016/j.iotech.2025.101041). Different doses (1, 3.33, 10, and 33 μg antigen/cap) of each antigen were tested. In the case of binding, the signal reached a plateau at 3.33 μg antigen/Cap (data not shown). Therefore this coating value was chosen for subsequent experiments. Anti-α3Gal IgE was detected in the three sera with all α3-galactosylated oligosaccharides, whereas none of the sera reacted with α4-galactosylated antigens or with unliganded streptavidin-coated Caps (PBS), demonstrating α3Gal-binding specificity (Supplementary Table S1, available at https://doi.org/10.1016/j.iotech.2025.101041). Regardless of the α3-galactosylated oligosaccharide, the highest concentration was always observed in the serum of patient 1. For two sera samples (patients 1 and 2), the binding to the α3Gal epitope in the polymeric form (70BP) was much higher than the binding to the α3Gal epitope in the monomeric form (70BM). However, the use of an alternative oligosaccharide with a terminal α3Gal connected to a lactose structure (Galβ4Glc) (10BP) instead of the ‘normal’ *N*-acetyllactosamine structure (Galβ4GlcNAc) (70BP) (Supplementary Figure S1, available at https://doi.org/10.1016/j.iotech.2025.101041), both being exposed to the same density (four oligosaccharides per PAA molecule), provided lower anti-α3Gal IgE concentrations for all three sera, demonstrating that the α3Gal epitope must be exposed in the correct oligosaccharide context to maximize IgE binding. Therefore 70BP at 3.33 μg antigen/Cap was selected for validation of our developed assay (hence referred to as the ‘70BP assay’) in larger cohorts of individuals and patients.

#### Description of the commercial bTG assay

The bTG assay was carried out using the ImmunoCAP 250 instrument (Thermo Fischer) and the appropriate reagents using an entirely automated procedure. The Caps were coated with bTG. This test has demonstrated a good correlation with Caps coated with cetuximab and α3Gal-conjugated human serum albumin (neoglycoconjugate) for red meat (beef) allergy. Using the same calibration curve, the fluorescence signal was converted into arbitrary units (kAU/l), with a detection threshold of 0.1 kAU/l.

### Statistical analysis

In both cohorts, age and total IgE levels were compared using the Mann–Whitney *U* test. ABO groups, sex, total IgE levels <150 or >150 kU/l, and Phadiatop results were compared using Fisher’s exact test.

The 0.1 kAU/l detection threshold was first used as the cut-off value to define a positive signal in both assays. receiver operating characteristic curves could not be used to determine the best threshold because of the low number of anaphylactic events in this cohort. An arbitrary threshold was determined by analyzing the figures.

The correlation between specific anti-α3Gal IgE levels measured using bTG and 70BP assays was determined using the Pearson linear correlation coefficient (*R*). The sensitivity, specificity, positive predictive value, and negative predictive value were calculated for both assays.

All tests were bilateral with an alpha risk of 5%. The tests were considered significant if the *P* value was <5%. Statistical tests were carried out using the R software version 3.2.2 (R Foundation, Vienna, Austria).

The data that support the findings of this study are available from the corresponding author upon reasonable request.

## Results

### Healthy blood donors

We applied both assays to sera from a cohort of blood donors to determine the prevalence of sera positive for anti-α3Gal IgE in our area (Table 1). Among the 299 blood donors, using the threshold of signal detection (0.1 kAU/l), 18 (6%) were found to be positive using the 70BP assay, 15 (5%) using the bTG assay, and 10 (3.3%) with both assays. The results of anti-α3Gal IgE with the two assays were well correlated (*r*^2^ = 0.91) (Figure 2). However, most positive sera were grouped into two distinct categories. Nine donors (3.1%) had concentrations higher than 15 times the signal detection threshold in both assays (range 2.32-4.81 for the 70BP assay and 1.72-2.71 for the bTG assay). The other positive donors usually had less than five times this threshold, with some discrepancies between the two assays (Figure 2). Indeed, in the range of 0.1-0.5 kAU/l of the 70BP assay, eight sera samples were detected positive with the bTG assay. In the range of 0.1-0.25 kAU/l of the bTG assay, three sera samples were detected as positive with the 70BP assay. Using thresholds of 0.5 kAU/l for the 70BP assay and 0.25 kAU/l for the bTG assay, respectively, and 6 (2.3%) and 4 (1.3%) of the 299 blood donors were found to be positive, respectively.

**Table 1:** Detection of anti-α3Gal IgE in a cohort of blood donors with our developed (70BP) and commercial (bTG) assays: the prevalence of anti-α3Gal IgE depending on age, sex, blood groups, and atopic markers (total IgE and Phadiatop) is presented

	Total	70BP assay			bTG assay		
		Positive	Negative	*P* value	Positive	Negative	*P* value
*n*	299	18	281	—	15	284	—
Mean age (years)	38.4	42.1	38.1	0.33	39.5	38.3	0.84
B or AB groups, *n*	39	1	38	0.49	0	39	0.23
O or A groups, *n*	260	17	243		15	245	
Sex, male/female, *n*	164/135	15/3	149/132	0.014	12/3	152/132	0.06
Mean total IgE	114.2	135.7	112.8	0.11	198.0	109.7	0.03
Total IgE >150/<150, *n*	61/238	4/14	57/224	0.77	6/9	55/229	0.09
Phadiatop +/−, *n*	96/203	7/11	89/192	0.60	3/12	93/191	0.4

bTG, bovine thyroglobulin; IgE, immunoglobulin E.

### Cancer patients

Among the 41 patients, four experienced an anaphylactic reaction during the first infusion of cetuximab (9.8%). One patient died 5 days after the infusion despite mechanical ventilation with oxygen (grade 5 severity). He was treated for metastatic disease, was an abusive alcohol and tobacco user, and was allergic to giblets. One patient experienced a grade 4 reaction (alive after the reaction but died during follow-up), and two patients experienced grade 3 reactions. Corticosteroids and antihistamine drugs were re-administered to all patients, and two patients (grades 5 and 4) required adrenaline infusion and hospitalization in the intensive care unit. Serum tryptase concentrations were available for three of the four patients, displaying very high levels of 90, 125, and 198 μg/l (normal range 0-11.4 μg/l), indicating mastocyte degranulation. Reintroduction of cetuximab was not allowed in the three patients who survived the anaphylactic reactions. A fifth patient showed a generalized rash (grade 2 reaction) with favorable evolution after corticosteroid and anti-H1 re-administration. Anaphylaxis is doubtful in this patient because cetuximab was continued without symptom recurrence. The remaining 36 patients in the cohort did not experience adverse reactions to cetuximab. The patient characteristics are reported in Supplementary Table S2, available at https://doi.org/10.1016/j.iotech.2025.101041, and the assay results are shown in Table 2.

**Table 2:** Detection of anti-α3Gal IgE in a cohort of 41 patients with head and neck cancer treated with cetuximab, using our developed (70BP) and commercial (bTG) assays

	Total	70BP assay			bTG assay		
		Positive	Negative	*P* value	Positive	Negative	*P* value
*n*	41	7	34	—	6	35	—
Mean age, years	63.0	57.4	64.2	0.05	59.3	63.7	0.18
Sex, male/female, *n*	36/5	7/0	29/5	0.57	6/0	30/5	1
Mean total IgE^a^	353.9	725.3	277.4	0.09	844.2	269.8	0.02
IgE >150/<150^a^, *n*	17/24	5/2	12/22	0.11	5/1	12/23	0.07
Phadiatop +/−, *n*	12/29	4/3	8/26	0.17	4/2	8/27	0.05

bTG, bovine thyroglobulin; IgE, immunoglobulin E.

a Dosage before the first cetuximab infusion.

**Figure 3:**
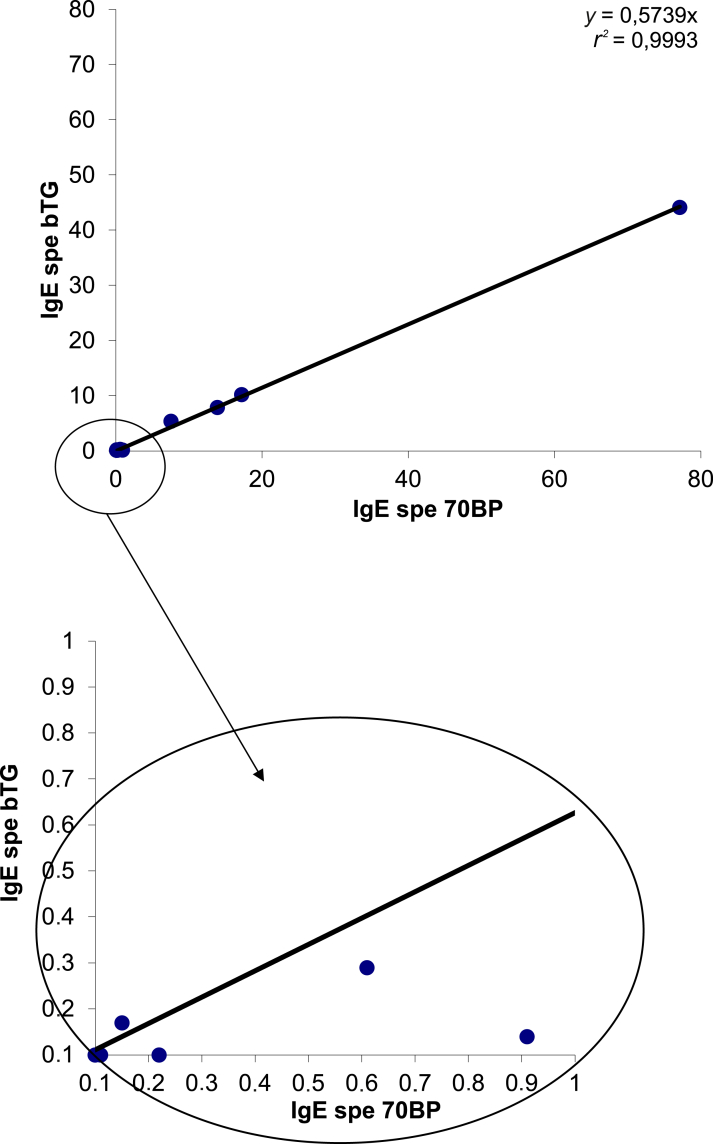
**Correlation between the two assays (70BP and bTG) to detect the presence of immunoglobulin E (IgE) in the 41 patients with cancer (*r***^**2**^**= 0.99).** bTG, bovine thyroglobulin.

Using the 70BP assay, the median signal value was 2.89 kAU/l (range 0-77.1 kAU/l) in the 41 patients and 28.94 kAU/l (range 7.55-77.1 kAU/l) in the 4 patients with anaphylactic reactions. Using the bTG assay, the median signal value was 1.66 kAU/l (range 0-44.1 kAU/l) in the 41 patients and 16.88 kAU/l (range 5.35-44.1 kAU/l) in the 4 patients with anaphylactic reactions. The patient with the cutaneous reaction had the following results: 0.61 kAU/l in the 70BP assay and 0.29 kAU/l in the bTG assay. No patient with a grade 3-5 clinical reaction had negative anti-α3Gal IgE in either assay (no false negatives). In this cohort of patients with cancer, the results of the two assays for the presence of α3Gal IgE were highly correlated (*r*^2^ = 0.99; Figure 3). Similar to blood donors, sera spread into two categories: those with values >50 times the signal detection threshold in both assays, corresponding to the four patients with anaphylaxis, and those just above the detection threshold in either of the two assays. Using the 0.1 kAU/l signal detection threshold, 7 (17.1%) patients with cancer were found to be positive using the 70BP assay and 6 (14.6%) using the bTG assay (Table 3). Using a 0.45 kAU/l threshold for the 70BP assay and a 0.25 kAU/l threshold for the bTG assay, 6 (14.6%) and 5 (12.2%) patients were positive, respectively. The only patient showing a discrepancy between the two assays did not experience any clinical reactions, suggesting that 0.9 kAU/l in the 70BP assay is a false-positive reaction. The patient with the grade 2 skin reaction was found to be very slightly positive for both assays (0.61 kAU/l for the 70BP assay and 0.29 kAU/l for the bTG assay).

**Table 3:** Repartition of positive and negative signals for both assays regarding anaphylactic reaction

	Grade 3-5 anaphylactic reaction, *n*	No anaphylactic reaction, *n*	Total, *n*		Grade 3-5 anaphylactic reaction, *n*	No anaphylactic reaction, *n*	Total, *n*
70BP +	4	3	7	bTG +	4	2	6
70BP −	0	34	34	bTG −	0	35	35
Total	4	37	41	Total	4	37	41

bTG, bovine thyroglobulin.

The predictive values of the 70BP and bTG assays for anaphylactic events (excluding the cutaneous reaction) were determined using the two possible thresholds. Regardless of the cut-off used, both assays had a sensitivity of 100% and a negative predictive value of 100%. Using 0.1 kAU/l as the signal detection threshold, the 70BP assay had a specificity of 91.9% and a positive predictive value of 57.1%, whereas the bTG assay had a specificity of 94.6% and a positive predictive value of 66.6%. The specificity and positive predictive value of 0.45 kAU/l for the 70BP assay were 94.6% and the positive predictive value was 66.6%. Using a cut-off of 0.25 kAU/l for the bTG assay, the specificity was 97.3% and the positive predictive value was 80%.

### Factors associated with the development of anti-α3Gal IgE antibodies

To search for individual factors predisposing to the development of anti-α3Gal IgE in the general population (blood donors), we analyzed the prevalence of serum positivity (using a cut-off of 0.1 kAU/l) according to available variables (Table 1): age, blood group, sex, and biological atopic markers (total IgE, more or less than 150 kU/l of total IgE, and Phadiatop test results). The blood group and age had no influence. Male sex was associated with the presence of αGal IgE in the 70BP assay, with a male-to-female sex ratio of 5 in favor of the male sex (*P* < 0.05), and a trend was observed with the bTG assay (sex ratio = 4; *P* = 0.06). Using the bTG assay, the other factors associated with the presence of αGal IgE were the mean total IgE concentration (*P* = 0.03) and an IgE level >150 kU/l (*P* = 0.09).

For patients with cancer, we searched for individual factors associated with the development of anti-αGal IgE by analyzing the distribution of the population with respect to age, sex, metastatic status, alcohol and tobacco consumption, clinical allergy, and atopic biological markers (Supplementary Table S2, available at https://doi.org/10.1016/j.iotech.2025.101041). The only factor associated with the risk of grade 3-5 clinical reactions was the total IgE concentration, which was higher in patients with anaphylactic reactions (*P* = 0.05). In the 70BP assay, the only factor associated with the presence of α3Gal IgE was age (*P* = 0.05). For the bTG assay, the factors associated with the presence of αGal IgE were total IgE (*P* = 0.02) and Phadiatop test positivity (*P* = 0.05). No significant influence of sex was found for either assay in the patient cohort, but all patients with anaphylactic reactions and positive signals were male (lack of power); female sex was poorly represented (only 5 patients out of 41). No significant influence of alcohol and tobacco was found in either assay; however, all patients with anaphylactic reactions and positive signals were alcohol and tobacco users.

## Discussion

Two assays were implemented to detect anti-α3Gal IgE and were tested in two cohorts to predict the risk of an anaphylactic reaction to cetuximab. Considering the signal threshold at 0.1 kAU/l, the prevalence of anti-αGal IgE was 5%-6% in healthy blood donors and 15%-17% in our cohort of treated patients, with a good correlation between both assays. The sensitivity and negative predictive value were 100% for both assays. The specificities were 91.9% and 94.6%, but the positive predictive values were 57.1% and 66.6% for the 70BP and bTG assays, respectively. After adjusting the cut-off values, the positive predictive values for anaphylaxis reached 66.6% and 80%, respectively, while maintaining a negative predictive value of 100%. High anti-α3Gal IgE concentrations were also shown to be associated with high total IgE levels and male sex, which could partly explain the higher incidence of anaphylactic reactions in patients with head and neck cancer than in those with colorectal cancer.

Both assays were designed to detect IgE against the α3Gal epitope. Binding to the α3Gal epitope in the polymeric form (70BP) was much higher than binding to the α3Gal epitope in a monomeric form (70BM), which could be explained by the higher density of α3Gal epitopes in the solid phase. bTG is also known to be heavily α-galactosylated, making this antigen particularly suitable for the detection of anti-α3Gal IgE. Indeed, the 70BP and commercial assays were well correlated in both a cohort of healthy blood donors and another cohort of patients with head and neck cancer treated with cetuximab.

Our first approach to interpreting the results was to consider the signal thresholds (0.1 kAU/l) provided by the manufacturer, based on the calibration curve, with the risk of detecting biologically sensitized patients not at risk of developing clinical manifestations. Indeed, the prevalence of sera positive for anti-α3Gal IgE was high, with many false-positive results, and the positive predictive values for anaphylactic reactions in both assays were far from satisfactory for use in clinical practice. The case of two particular sera (0.36 with bTG/0.49 with 70BP on one side and <0.1 with bTG/0.49 with 70BP on the other) illustrates that the 70BP assay is less discriminant than the bTG assay. Altogether, these data suggest that signals <0.25 for the bTG assay and 0.5 for the 70BP assay should be regarded with caution in terms of clinical relevance. The limited size of the cohort did not allow us to elaborate on a receiver operating characteristic curve and calculate the ideal thresholds. However, it graphically appeared that most, if not all, false-positive results displayed low levels in both assays, and it was easy to determine arbitrary thresholds, that is, 0.45 kAU/l for the 70BP assay and 0.25 kAU/l for the bTG assay. Using these new thresholds, the positive predictive value increased from 57.1% to 66.6% for the 70BP assay, and from 66.6% to 80% for the bTG assay (commercial assay). Remarkably, modifying these thresholds did not alter the negative predictive value of either assay, which remained 100%. Altogether, the bTG assay provided the best negative and positive predictive values, rendering it very useful in clinical practice.

With the 0.25 kAU/l cut-off value in the bTG assay, the prevalence of positive sera was 1.3% in blood donors and 12.2% in treated patients. The incidence of hypersensitivity reactions to cetuximab varies (3%-22% of patients), depending on the country (higher in the United States) and the type of cancer. Although blood group B and AB donors constitutively express a fucosylated α3Gal epitope in their tissues and could be at a lower risk of developing anti-α3Gal IgE antibodies, the ABO blood group does not seem to influence the development of α3Gal IgE. The incidence of head and neck cancer is higher than that of other cancers without a specific explanation. The only individual factor found in both assays to be associated with the presence of α3Gal IgE in blood donors was male sex, with an impressive ratio of >4 in favor of males. This could not be evaluated in the patient cohort, where the male sex is overrepresented, as is usually seen in head and neck cancer. The influence of sex on allergy is debated, with a slightly higher prevalence of male sex in childhood and a slightly higher incidence of female sex in adulthood. To the best of our knowledge, this is the first time that sensitization to a specific allergen has been shown to differ greatly between males and females, excluding some occupational allergies in professions characterized by a nonequilibrated sex ratio. Exposure to ticks, meat, or giblets does not differ significantly between male and female blood donors. Based on our current knowledge of anti-α3Gal sensitization, the higher incidence of anti-α3Gal IgE in male patients is difficult to explain. This aligns with the higher incidence of allergic reactions observed in patients with head and neck cancer receiving cetuximab than in patients with colon cancer. Moreover, besides sex, an additional explanation for this higher incidence in patients with head and neck cancer could be that these patients frequently have higher IgE levels. Indeed, the concentrations of anti-α3Gal IgE were correlated with those of total IgE, particularly with the bTG assay, and to a lesser extent with Phadiatop. It is unlikely that these associations result from anti-α3Gal non-specific signals in the presence of high levels of IgE because it has already been shown that anaphylactic reactions to cetuximab are more frequent in patients with atopy, who in fact have high IgE levels and a positive Phadiatop.

In this cohort of patients with precise clinical data regarding cetuximab infusion, we found a 9.8% risk of anaphylactic reaction after the first infusion of cetuximab, including one grade 5. In our experience, the risk of a fatal outcome from anaphylaxis can be prevented by close monitoring of patients during the first infusion but is not entirely sufficient for safety. Once again, we wish to warn oncologists about the possible fatal outcomes of the first cetuximab infusion and continue to inform them about the existence of predictive assays to secure their practice. Indeed, the detection of preexisting anti-α3Gal IgE is a major issue because of the wide use of cetuximab, particularly in locally advanced, recurrent, or metastatic head and neck cancer, where no effective alternative is possible. Until now, the described assays had good sensitivity and a good negative predictive value, but most of them had a poor positive predictive value, with the risk of overestimation, raising unnecessary concerns, and even loss of opportunity if cetuximab is unnecessarily contraindicated. The bTG assay, which is commercially available worldwide, meets this demand. The European Medicines Agency (EMA), but not the United States Food and Drug Administration (FDA), has recommended the detection of anti-cetuximab IgE before the first infusion; it would be more precise to refer to anti-α3Gal IgE in accordance with their specificity and the available methods of detection. Additional studies in larger cohorts of patients with cancer (head, neck, and colorectal cancer) are now required to more precisely define the clinically relevant cut-off value of this assay and to analyze its cost-effectiveness.
