# Supplementary material for: Clinical usefulness of anti-α3Gal immunoglobulin E assays for cetuximab-mediated anaphylaxis in head and neck cancer
Source: Immunooncol Technol. 2025 Feb 7;25:101041. doi: 10.1016/j.iotech.2025.101041 (PMC11919289; doi:10.1016/j.iotech.2025.101041)
Supplement: Supplementary Material [file mmc1.docx]

Table S1. Selection of the best α-galactosylated antigen in our developed assay*.

|  | **70BM** / 8BM | **70BP** / 8BP | **10BP** / 37BP | PBS |
| --- | --- | --- | --- | --- |
| Serum 1 | **1.26** / <0.10 | **3.17** / <0.10 | **1.42** / <0.10 | <0.10 |
| Serum 2 | **0.27** / <0.10 | **0.76** / <0.10 | **0.39** / <0.10 | <0.10 |
| Serum 3 | **0.37** / <0.10 | **0.37** / <0.10 | **0.35** / <0.10 | <0.10 |

*Concentrations measured for three representative sera with the three antigen pairs described in Figure 2.


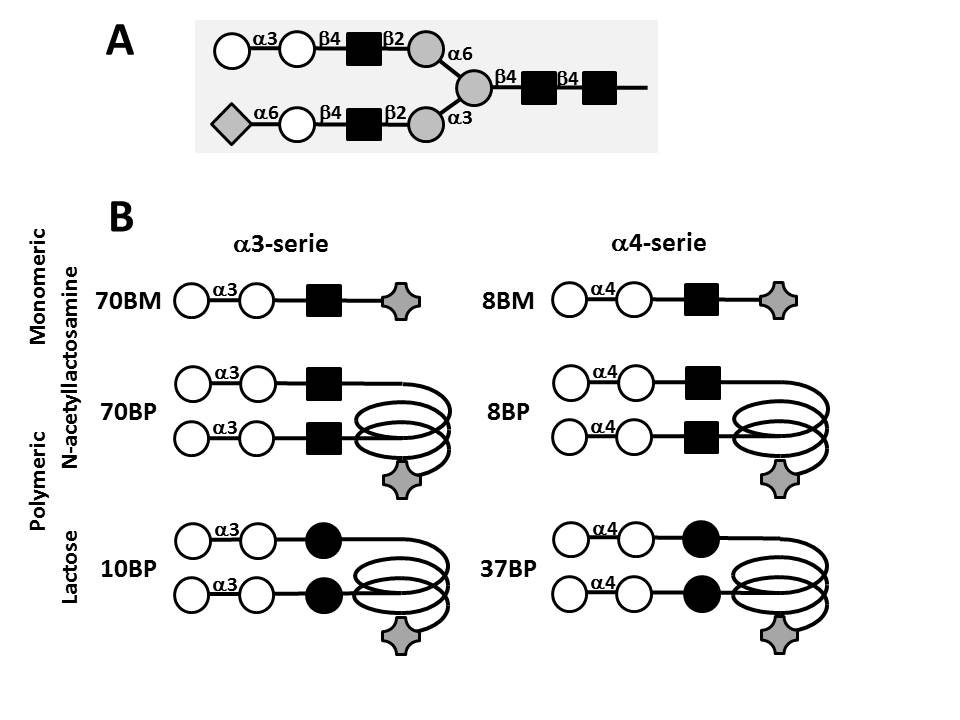


Figure S1. **Schematic representation of a typical biantennary *N*-glycan from cetuximab Fab or bovine thyroglobulin (A), and oligosaccharides initially tested to set up our developed assay (B).**

Table S2. Cancer patient characteristics.

|  | **Total** | Grade 3-5 anaphylactic reaction | No anaphylactic reaction | p |
| --- | --- | --- | --- | --- |
| Number | 41 | 4 | 37 | - |
| Mean age | 63.6 | 56.4 | 63.8 | 0.11 |
| Gender Male/Female | 36/5 | 4/0 | 32/5 | 1 |
| Metastasis: Yes/No | 18/23 | 2/2 | 16/21 | 1 |
| Alcohol: Yes/No | 29/12 | 4/0 | 25/12 | 0.30 |
| Tobacco: Yes/No | 31/10 | 4/0 | 27/10 | 0.56 |
| Allergy: Yes/No | 11/30 | 1/3 | 10/27 | 1 |
| Mean total IgE* | 353.9 | 1042.5 | 279.4 | 0.05 |
| Total IgE>150/<150* | 17/24 | 3/1 | 14/23 | 0.29 |
| Phadiatop^®^+/- | 12/29 | 3/1 | 9/28 | 0.07 |

* Dosage before cetuximab first infusion.
